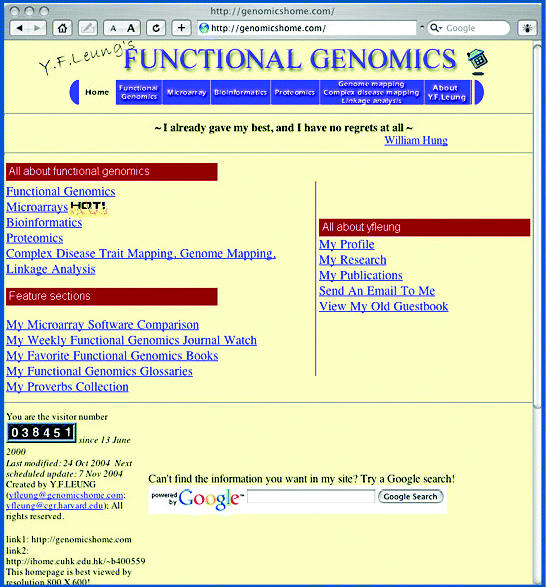# TXGnet: Y.F. Leung’s Functional Genomics

**Published:** 2004-11

**Authors:** Erin E. Dooley

With its sequencing completed in 2003, scientists set their sights on determining the basic structure and inner workings of the human genome. This movement has spawned numerous new scientific specialties that have been supported by the growth of data-generating technologies. One of these interdisciplinary fields, functional genomics, is devoted to linking gene expression to function (or dysfunction) in cells, organs, and tissue. On his website titled Y.F. Leung’s Functional Genomics, located at **http://genomicshome.com/**, Harvard researcher Yuk Fai Leung sketches out the current state of this new field of study.

The homepage of the site is divided into three central sections. The main section, titled All About Functional Genomics, is an assemblage of links to relevant outside resources such as “omics” glossaries and the Department of Energy Genomes to Life program. Also in this section are pages of resources for related fields including bioinformatics and proteomics. Leung has also brought together resources on the use of chaos and nonlinear dynamics in genomics, and on the ethical, legal, and social issues surrounding genomics. A group of links to institutes and core facilities conducting functional genomics work is also provided.

Microarray technology has been crucial to the development of functional genomics. The Microarrays subsection gives an overview of what exactly these tools are, as described through videos, technology reviews, even cartoons, and provides descriptions of all of the various equipment and technology required to perform this sort of analysis. The Language & Standard subpage lists links to resources on communicating results with others within the discipline. Listings of relevant courses, video seminars, conferences, and workshops are also available.

The Bioinformatics subsection of the website contains links to more than 50 databases. This subsection, like the Microarrays subsection, also has pages devoted to the language and algorithms used in bioinformatics as well as data standardization. The Ontology page has links to resources on the efforts to develop a standard, universal vocabulary that can be used across the “omics” fields for all organisms.

Other subsections are devoted to proteomics and to genome mapping, complex disease mapping, and linkage analysis. They are populated much as the other two subsections, with pages of glossaries, educational opportunities, calendars, and the like.

This website also has a novel Feature Sections element that bench scientists will find useful. Leung has put together a microarray software comparison featuring 13 primary types of software, including programs for data preprocessing, analysis, and annotation. The page for each software type has a definition of the software’s use, suggested readings, and lists of the products available in each category. Also on offer is a compendium of peer-reviewed journal articles related to functional genomics, including a list of biotech business articles. Leung also provides a reading list of books on functional genomics topics and an in-depth functional genomics glossary.

## Figures and Tables

**Figure:**